# Prevalence of Childhood and Adolescent Overweight and Obesity from 2003 to 2010 in an Integrated Health Care Delivery System

**DOI:** 10.1155/2013/417907

**Published:** 2013-07-18

**Authors:** Scott Gee, Debbie Chin, Lynn Ackerson, Dewey Woo, Amanda Howell

**Affiliations:** ^1^Prevention & Health Information, Regional Health Education, The Permanente Medical Group, Inc., 1950 Franklin Street, 13th Floor, Oakland, CA 94612, USA; ^2^The Permanente Medical Group, Inc., Oakland, CA 94612, USA; ^3^Kaiser Permanente Division of Research, USA

## Abstract

An observational study of the Kaiser Permanente Northern California (KPNC) BMI coding distributions was conducted to ascertain the trends in overweight and obesity prevalence among KPNC members aged 2–19 between the periods of 2003–2005 and 2009-2010. A decrease in the prevalence of overweight (−11.1% change) and obesity (−3.6% change) and an increase in the prevalence of healthy weight (+2.7% change) were demonstrated. Children aged 2–5 had the greatest improvement in obesity prevalence (−11.5% change). Adolescents aged 12–19 were the only age group to not show a decrease in obesity prevalence. Of the racial and ethnic groups, Hispanics/Latinos had the highest prevalence of obesity across all age groups. The KPNC prevalence of overweight and obesity compares favorably to external benchmarks, although differences in methodologies limit our ability to draw conclusions. Physician counseling as well as weight management programs and sociodemographic factors may have contributed to the overall improvements in BMI in the KPNC population. Physician training, practice tools, automated BMI reminders and performance feedback improved the frequency and quality of physician counseling. BMI screening and counseling at urgent visits, in addition to well-child care visits, increased the reach and dose of physician counseling.

## 1. Introduction

From 1970 to 2000, the number of obese children in the USA tripled. From 2000 to 2010, no statistically significant linear trends in body mass index (BMI) were detected; however, 30.4% of children and adolescents aged 2 through 19 years were overweight or obese in 2009-2010 [1]. Obese children are at increased risk for cardiovascular disease, type 2 diabetes, and other health conditions [2]. As a consequence of the expected increase in chronic conditions, some experts are predicting a shortened lifespan for this generation of America's youth compared with that of their parents [3]. Obesity and the associated risks, including stigma, can also result in a lower quality of life [4]. Hospital costs for children with conditions caused or worsened by obesity have increased [5]. Overweight children often grow up to be overweight adults [6], and the medical care costs of adult obesity in the United States were estimated to be as much as $147 billion in 2006 [7]. Including lost productivity, obesity costs California $21 billion each year [8].

Although some studies suggest that child obesity is less prevalent in California than in other states [9, 10], BMI data collected in California public schools reveals great disparities in obesity prevalence and trends in different counties. A study looking at BMI trends in California schools from 2001 to 2008 found a declining prevalence of obesity in some populations, but not in others [11]. Another study looked at BMI trends in California schools from 2005 to 2010. The prevalence of overweight and obese children declined by 1.1% from 2005 to 2010; however, the prevalence in many Northern California counties served by Kaiser Permanente Northern California (KPNC) increased over that time period [12]. 

**Figure 1:**
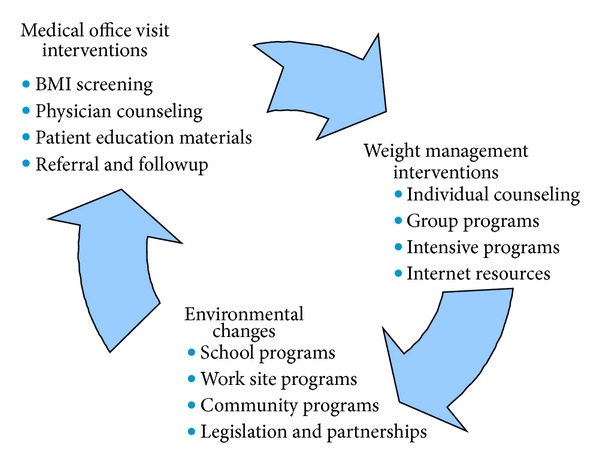
KPNC's approach to childhood obesity.

In 2001, KPNC began a multifaceted initiative to address childhood obesity in Northern California. This initiative had 3 components: medical office visit interventions, weight management interventions, and environmental changes (Figure 1) [13]. We initiated our study to examine the effect of these approaches on the change in obesity prevalence in the pediatric population of KPNC. To this end, we examined changes in BMI among KPNC members aged 2–19 from 2003–2005 to 2009-2010. The study had the following goals.

1. Identify existing patterns amongst age and racial/ethnic groups.
2. Assess progress towards reversing the childhood obesity epidemic.
3. Identify areas or population segments to target for future interventions.

## 2. Methods

KPNC is an integrated, prepaid, nonprofit health care delivery system in Northern California. KPNC has 3.3 million members, which represents 35–40% of the insured market in the Northern California catchment area. The median income of the membership parallels that of the general population, with fewer members at both the high and low extremes [14, 15]. Distributions of BMI category prevalence were calculated on 2 sets of cross-sectional aggregate data (2003–2005 and 2009-2010, *n* = 254, 007 and *n* = 426, 667, resp., e.g., size comparability between the two cohorts). The population in this study included Medi-Cal and Kaiser Permanente health plan members who had a visit with either their family medicine or pediatric provider. Age, sex, race/ethnicity, and pediatric BMI category (BMI percentile <5, 5–84, 85–94.9, and ≥95) were collected for each member. Sex and race/ethnicity were self-reported by members and entered into HealthConnect, Kaiser Permanente's national electronic health record (EHR). Race/ethnicity categories included Asian, Black, Hispanic/Latino, White, or Other. If no value was entered or the patient declined to state, the variable was marked as “unknown.”

In 2003–2005, BMI was captured primarily at well-child visits and coded in the EHR, whereas in 2009-2010, BMI was captured at well-child and urgent care visits and coded in the EHR. Young adults aged 18 and 19 were included to be consistent with the National Health and Nutrition Examination Survey (NHANES) analysis [1]. 

To compare distributions of BMI categories between the time periods, races, and/or age groups, it was necessary to adjust the distributions for sample comparability. For example, differences in the prevalence rates of obesity at two different times could be due to the programs implemented between the two times, temporal trends in the community, or differences in the characteristics of the sample of people at the two time points. To account for differences in the characteristics of the samples, prevalence rates were directly adjusted for age, gender, and race/ethnicity [15]. The reference population was all children from both time periods and from all service areas. Because the rates in each study group are applied to the same reference population, the predicted prevalence rates were not affected by differences in demographic distributions over time, thus ensuring comparability over time and between age and race/ethnicity groups. 

To compare the rates of obesity and overweight within KPNC to those in the surrounding regions, we used prevalence rates amongst 5th, 7th, and 9th graders from the counties of Alameda, Contra Costa, Fresno, Madera, Marin, Merced, Sacramento, San Francisco, San Joaquin, San Mateo, Santa Clara, Solano, Sonoma, Stanislaus, and Yolo in 2005 and 2010 [12]. Given the geographic mapping of KPNC service areas, not all service areas are captured via these listed counties. The KPNC sample for this comparison consisted of members aged 6–19 in 2003–2005 and in 2009-2010. 

To test for statistically significant differences over time (2003–2005 period versus 2009-2010 period), a set of 4 logistic regression models were fit, one for each BMI category. In each model, the independent variables were age, sex, race/ethnicity, time (2003–2005 versus 2009-2010), and the interactions between time and each of the other variables. When an interaction was statistically significant (*P* < 0.05), results could not be interpreted for either of the main effects in the interaction. In these cases, the models were refit, stratifying on one of the statistically significant interaction variables. The variable of the most interest in this report is change over time. The stratified model was tested for a time effect within each combination of age and race. Results are reported as odds ratios (ORs) with their 95% confidence intervals (CI). 

This study was done as a quality improvement effort to inform KPNC's approach to reduce the prevalence of overweight and obesity in its pediatric population and therefore did not require IRB review.

## 3. Results

### 3.1. Demographics

The samples from the 2 study periods were similar with regard to age and sex distribution (Table 1). However, the 2009-2010 sample had a higher prevalence of Hispanics/Latinos.

### 3.2. Prevalence by Weight Category

We compared the directly adjusted BMI category prevalence rates for the 2 study periods (Table 2). Overall, encouraging patterns have emerged, with improvements in the proportion of the population of healthy weight (+2.7% change) and a decrease in the proportion who are overweight (−11.1% change). The prevalence of obesity decreased regionwide (−3.6% change) and among all age groups except for those ages 12–19 years (1.6% change). Children aged 2–5 had the greatest improvement in obesity (−11.5% change). An increase in the underweight category was observed in all age groups, but the prevalence is low, and further analysis is needed to inform the discussion.

BMI category prevalence rates were directly adjusted for each combination of age and race/ethnicity separately for the 2 time periods (Table 3). Of the racial and ethnic groups, Hispanics/Latinos had the highest prevalence of obesity across all age groups.

All age groups and races showed a statistically significant decrease (OR > 1) in overweight prevalence. Asians, whites, and unknown races aged 2–11 and other races aged 2–5 showed a statistically significant decrease (OR > 1) in obesity prevalence.

### 3.3. External Benchmarking

In 2009-2010, childhood overweight and obesity prevalence rates in KPNC were lower than national prevalence rates for children aged 2–19 [1] (Table 4).

Obesity is more prevalent in KPNC Hispanics and Latinos aged 2–19 than it is in a national cohort [1] (Table 5). Obesity is less prevalent in KPNC blacks and whites than in the national cohort.

The overall overweight and obesity prevalence rates in KPNC for ages 6–19 years compare favorably with the overweight and obesity prevalence rates for school-age children in the counties served by KPNC [12] (Table 6). However, differences in methodology limit our ability to draw conclusions.

## 4. Discussion

In the pediatric population of KPNC, obesity and overweight decreased between 2003 and 2010, in contrast to comparator populations in the same geographic location and nationwide. Although our datasets captured only those members who came to a KPNC facility for a visit during the time periods 2003–2005 and 2009-2010, the sample sizes (*n* > 200,000 for each cohort) are sufficiently large to allow these data to be generalized to the KPNC pediatric population. 

Improvements in overweight and obesity among KPNC members may be attributable to sociodemographic differences and/or differences in the clinical care received by KPNC members versus nonmembers. The KPNC adult membership does not significantly differ from the adult population of Northern California with regard to age, sex, or race/ethnicity. However, compared to nonmembers in Northern California, the KPNC adult membership does have significantly lower percentages of men and women with household incomes <200% above the federal poverty line, with incomes of ≤$25,000, and who have not graduated from high school [14]. Families with low income and low educational attainment are more likely to have overweight children [16, 17]. The KPNC adult population has a significantly higher percentage of people who are employed at least 20 hours/week compared to the Northern California population, for both men and women. Several studies have found that a child is more likely to be overweight if his or her mother worked more hours per week over the child's life [18, 19]. 

**Figure 2:**
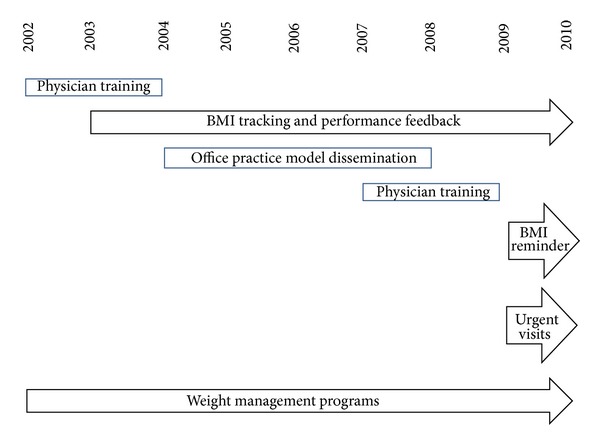
KPNC strategies to address childhood obesity.

KPNC members may have received clinical care that nonmembers did not receive. From 2002 to 2004, training was provided for all KPNC pediatricians and family practitioners to measure BMI and provide family-centered nutrition and physical activity counseling at well-child care visits (Figure 2). Training occurred over multiple sessions and was provided at the clinic and by teleconference. Supplemental training on motivational interviewing was also available in group sessions and online. Medical assistants were trained to measure and plot BMIs on growth charts showing BMI percentile for age. Office system tools to support BMI screening and counseling, including BMI wheel calculators, exam room posters, and patient education materials, were provided at no charge to clinics. The exam room poster had 4 key messages and was used to provide family education as well as to facilitate physician counseling. In 2003, electronic data collection of BMI category began, and departments received feedback on their rates of BMI measurement at well-child visits. Provider counseling on BMI and associated health risks may influence a parent's perception of the child's weight as unhealthy and may potentially increase parent readiness to take action [20, 21]. For obese patients, providers were encouraged to arrange for a follow-up visit to provide more intensive family-centered counseling and to review lab test results if indicated. Follow-up visits with physicians and dieticians have demonstrated modest improvements in BMI [22, 23]. By 2005, BMI screening and physician counseling for nutrition and physical activity were provided at over 90% of KPNC well-child care visits, and this performance was maintained through 2010. BMI screening was tracked using visit coded BMI category diagnosis. Outside of KPNC, BMI screening and counseling were provided much less frequently [24–26]. From 2004 to 2008, the KPNC office practice model was disseminated nationally to all of the other Kaiser Permanente regions as well as other health systems. The dissemination included practice tools as well as physician training.

From 2002 to 2006, KPNC tripled the number of facilities offering weight management programs for families. Self-care materials, web-based programs, and single-session weight management programs were offered in all service areas at no additional cost to members. Multisession weight management programs were also offered in the clinic setting and varied in intensity from 2 to 20 sessions. These programs were led by health educators and focused on family health behavior change. Many of these programs were evaluated, with most programs demonstrating improvements in health behaviors and multisession programs yielding modest improvements in weight. Many weight management programs for families have produced modest improvements in BMI, with more intensive programs yielding greater improvements [27]. Although the multisession weight management programs led to modest improvements in BMI among attendees, the number of families who attended these programs was relatively small compared to the number of obese children in the KPNC membership.

Pediatricians and family practitioners received training and tools to implement the “Expert Committee Recommendations Regarding the Prevention, Assessment, and Treatment of Child and Adolescent Overweight and Obesity” in 2007–2009 [28]. The training was provided by teleconference as well as at the clinic. 

In 2009, an EHR was fully implemented and a reminder system was developed that provided all members and providers with an annual BMI screening reminder that members could view on the visit receipt at an office visit and online. Personalized BMI information and education were developed for the After Visit Summary, a printed summary of the visit provided to patients. The use of the EHR to facilitate BMI screening and counseling was demonstrated in a study from Kaiser Permanente Southern California (KPSC) [29]. KPSC used an automated alert for overweight or obese patients to prompt providers to code an obesity diagnosis, provide counseling, and order appropriate lab tests. BMI measurement, coding for a diagnosis of obesity at an office visit, and counseling all significantly increased from 2007 to 2010. The prevalence of obesity over the study period was stable, but not improved, with 17% overweight, 14% moderately obese, and 7% to 9% extremely obese [29]. KPNC used a similar approach to improve BMI screening and counseling, but demonstrated a decrease in overweight and obesity prevalence. A longer study period and more intensive training for providers may have contributed to these differences. However, including only the BMI category, as opposed to the continuous BMI measurement, does reduce the precision of the inferences we can make.

Beginning in 2009, providers were encouraged to measure BMI and provide counseling annually at urgent care visits in addition to well-child visits. Clinics were provided quarterly feedback on BMI screening and counseling performance. The addition of BMI screening at urgent visits increased BMI data capture, provided counseling for patients who did not have a well-child care visit, and increased the likelihood that a patient would receive more than one counseling session during the calendar year. Since Medicaid-eligible children are less likely to be compliant with well-child care, more high risk children had BMI screening and received counseling [30]. Training combined with performance feedback can improve BMI screening by physicians [31]. In 2009, the Healthcare Effectiveness Data and Information Set (HEDIS) added annual BMI screening, nutrition counseling, and physical activity counseling for children aged 3–17 [32]. KPNC demonstrated improvement for all 3 HEDIS measures from 2009 to 2010. 

Improvement in the prevalence of obesity among KPNC members 2–5 years is encouraging and mirrors improvements in obesity prevalence among low-income preschool-aged children [33]. Despite the overall improvements, several groups showed less improvement over the period examined. Obese adolescents and obese Hispanics/Latinos aged 6–19 in particular may need further intervention. 

## 5. Conclusion

Findings from this observational study highlight the strides that KPNC has made in addressing obesity among children and adolescents in its member population. Although weight management programs and sociodemographic factors may have contributed to the overall improvements in BMI in the KPNC population, the contribution was likely to be small. Most of the improvements were likely attributable to physician counseling. Physician training, practice tools, automated BMI reminders, and performance feedback improved the frequency and quality of physician counseling. BMI screening and counseling at urgent visits, in addition to well-child care visits, increased the reach and dose of physician counseling.

These findings have also shed light on the varied response to the intervention. Understanding this variation will be key to quality improvement and will inform the development of strategies and interventions to effectively target groups at risk and to decrease obesity prevalence rates among KPNC's pediatric population. Two populations that will need further intervention and research are obese adolescents and obese Hispanics/Latinos aged 6–19.

## Figures and Tables

**Table 1:** Age, sex, and race/ethnicity distributions at each time period.

	2003–2005	2009-2010
	*n* (%)	*n* (%)
Ages, years		
02–05	85,804 (33.8%)	137,479 (32.2%)
06–11	82,464 (32.5%)	140,815 (33.0%)
12–19	85,739 (33.7%)	148,383 (34.8%)
Sex		
Male	128,598 (50.6%)	218,249 (51.1%)
Female	125,404 (49.4%)	208,423 (48.9%)
Race/ethnicity		
Asian	34,413 (13.6%)	63,947 (15.0%)
Black	23,179 (9.1%)	34,175 (8.0%)
Hispanic/Latino	25,959 (10.2%)	55,977 (13.1%)
White	98,449 (38.8%)	153,714 (36.3%)
Other	21,660 (8.5%)	40,918 (9.6%)
Unknown	50,342 (19.8%)	76,941 (18.0%)

Total	254,007	426,677

**Table 2:** Directly adjusted BMI category distribution by weight category, age, and time.

	Underweight (<5th percentile)				Healthy weight (5–84.9th percentile)				Overweight (85–94.9th percentile)				Obese (≥95th percentile)			
	2003–2005^a^	2009-2010^a^	Percentage point difference	Percent change^b^	2003–2005^a^	2009-2010^a^	Percentage point difference	Percent change^b^	2003–2005^a^	2009-2010^a^	Percentage point difference	Percent change^b^	2003–2005^a^	2009-2010^a^	Percentage point difference	Percent change^b^
Ages 02–05 *N* = 223,279	2.2% (2.1, 2.3)	2.9% (2.8, 3.0)	**+0.7**	**+31.8%**	75.5% (75.2, 75.8)	77.3% (77.1, 77.6)	**+1.8**	**+2.4%**	11.0% (10.8, 11.2)	9.7% (9.5, 9.8)	**−1.3**	**−11.8%**	11.3% (11.1, 11.5)	10.0% (9.9, 10.2)	**−1.3**	**−11.5%**
Ages 06–11 *N* = 223,275	1.5% (1.4, 1.6)	1.8% (1.8, 1.9)	**+0.3**	**+20.0%**	62.3% (61.9, 62.6)	65.1% (64.8, 65.3)	**+2.8**	**+4.5%**	15.9% (15.7, 16.2)	13.8% (13.6, 14.0)	**−2.1**	**−13.2%**	20.3% (20.0, 20.6)	19.3% (19.1, 19.5)	**−1.0**	**−4.9%**
Ages 12–19 *N* = 234,120	1.5% (1.4, 1.5)	1.6% (1.5, 1.7)	+0.1	+6.7%	63.2% (62.9, 63.5)	64.3% (64.1, 64.6)	**+1.1**	**+1.7%**	16.4% (16.1, 16.6)	14.8% (14.6, 15.0)	**−1.6**	**−9.8%**	19.0% (18.8, 19.3)	19.3% (19.1, 19.5)	+0.3	1.6%
All *N* = 680,674	1.7% (1.7, 1.8)	2.1% (2.1, 2.2)	**+0.4**	**+23.5%**	67.0% (66.7, 67.1)	68.8% (68.7, 60.0)	**+1.8**	**+2.7%**	14.4% (14.3, 14.6)	12.8% (12.7, 12.9)	**−1.6**	**−11.1%**	16.9% (16.8, 17.1)	16.3% (16.1, 16.4)	**−0.6**	**−3.6%**

^a^Values are shown as mean (95% CI).

^b^Percent change = percentage point difference/percent weight category in 2003–2005.

Bold indicate statistically significant (*P* < 0.05) time effects.

**Table 3:** Directly adjusted BMI category distribution by weight category, age, race/ethnicity, and time.

	Percent overweight			Percent obese		
	BMI 85–94.9th percentile			BMI ≥ 95th percentile		
	2003–2005^a^	2009-2010^a^	OR (95% CI)	2003–2005^a^	2009-2010^a^	OR (95% CI)
Asian						
Ages 02–05	8.6% (8.2, 9.1)	7.7% (7.4, 8.1)	**1.13 (1.04, 1.22) **	9.6% (9.1, 10.1)	8.1% (7.8, 8.4)	**1.21 (1.12, 1.30)**
Ages 06–11	15.0% (14.3, 15.7)	12.8% (12.3, 13.3)	**1.20 (1.13, 1.29)**	17.3% (16.6, 18.0)	15.3% (14.8, 15.8)	**1.17 (1.10, 1.24)**
Ages 12–19	15.0% (14.3, 15.7)	13.7% (13.2, 14.2)	**1.11 (1.03, 1.18) **	14.1% (13.4, 14.7)	13.8% (13.3, 14.3)	1.00 (0.94, 1.08)
Black						
Ages 02–05	12.5% (11.7, 13.3)	10.5% (9.9, 11.1)	**1.22 (1.11, 1.34)**	13.0% (12.2, 13.8)	12.2% (11.6, 12.9)	1.08 (0.98, 1.18)
Ages 06–11	16.4% (15.5, 17.2)	14.6% (14.0, 15.3)	**1.15 (1.06, 1.24) **	24.2% (23.2, 25.1)	24.4% (23.6, 25.2)	0.99 (0.93, 1.06)
Ages 12–19	17.6% (16.8, 18.4)	16.2% (15.5, 16.8)	**1.11 (1.03, 1.19) **	24.4% (23.5, 25.3)	25.1% (24.4, 25.9)	0.96 (0.90, 1.03)
Hispanic/Latino						
Ages 02–05	13.6% (12.8, 14.4)	11.9% (11.4, 12.4)	**1.17 (1.08, 1.27) **	16.7% (15.8, 17.5)	16.0% (15.4, 16.6)	1.05 (0.97, 1.13)
Ages 06–11	18.4% (17.6, 19.2)	15.4% (14.9, 15.9)	**1.24 (1.16, 1.32) **	28.1% (27.3, 29.0)	29.0% (28.4, 29.7)	0.95 (0.90, 1.01)
Ages 12–19	18.3% (17.5, 19.1)	17.0% (16.5, 17.5)	**1.10 (1.03, 1.17) **	25.5% (24.6, 26.4)	26.3% (25.7, 26.9)	0.95 (0.90, 1.01)
White						
Ages 02–05	10.8% (10.5, 11.2)	9.4% (9.1, 9.6)	**1.18 (1.12, 1.23)**	8.8% (8.5, 9.1)	7.7% (7.4, 7.9)	**1.16 (1.11, 1.22) **
Ages 06–11	15.2% (14.8, 15.6)	13.3% (13.0, 13.6)	**1.17 (1.12, 1.22) **	16.5% (16.1, 17.0)	14.9% (14.6, 15.2)	**1.13 (1.09, 1.18) **
Ages 12–19	15.4% (15.1, 15.8)	14.1% (13.8, 14.4)	**1.12 (1.08, 1.16) **	15.5% (15.2, 15.9)	15.8% (15.5, 16.1)	0.97 (0.94, 1.01)
Other						
Ages 02–05	12.1% (11.4, 12.7)	10.9% (10.4, 11.4)	**1.12 (1.04, 1.21) **	15.0% (14.4, 15.7)	14.1% (13.5, 14.6)	**1.08 (1.01, 1.16) **
Ages 06–11	17.1% (16.2, 18.1)	14.6% (14.0, 15.2)	**1.21 (1.12, 1.31) **	25.1% (23.0, 26.2)	25.0% (24.3, 25.7)	1.01 (0.94, 1.08)
Ages 12–19	18.3% (17.3, 19.4)	15.4% (14.7, 16.1)	**1.24 (1.14, 1.36) **	26.4% (25.2, 27.6)	26.2% (25.4, 27.1)	1.00 (0.93, 1.08)
Unknown						
Ages 02–05	11.0% (10.5, 11.5)	9.8% (9.4, 10.2)	**1.13 (1.06, 1.21) **	12.1% (11.6, 12.6)	10.1% (9.7, 10.5)	**1.23 (1.15, 1.31) **
Ages 06–11	15.7% (15.1, 16.2)	13.8% (13.4, 14.2)	**1.17 (1.10, 1.23) **	20.2% (19.6, 20.8)	18.8% (18.4, 19.3)	**1.10 (1.05, 1.15) **
Ages 12–19	16.4% (15.9, 17.0)	14.5% (14.1, 14.9)	**1.15 (1.09, 1.21) **	19.5% (18.9, 20.1)	19.6% (19.2, 20.1)	1.00 (0.95, 1.05)

^a^Values are shown as rate (95% CI).

Bold indicate statistically significant (*P* < 0.05) time effects.

**Table 4:** Overweight and obesity in children aged 2–19, in KPNC and NHANES.

	NHANES 2009-2010^a^	KPNC 2009-2010
Overweight and obese (BMI ≥ 85th percentile)	31.8%	29.2%
Obese (BMI ≥ 95th percentile)	16.9%	16.4%

^a^Data taken from reference [1].

**Table 5:** Comparison of obesity in children aged 2–19 years in the NHANES and KPNC populations in 2009-2010, stratified by race and gender.

	NHANES PEDIATRIC BMI ≥ 95th percentile %	KPNC PEDIATRIC BMI ≥ 95th percentile %
Both sexes		
White	14.0%	12.8%
Black	24.3%	21.0%
Hispanic/Latinos	21.2%	24.6%
Male		
White	16.1%	13.8%
Black	24.3%	19.4%
Hispanic/Latinos	23.4%	27.1%
Females		
White	11.7%	11.6%
Black	24.3%	22.7%
Hispanic/Latinos	18.9%	22.1%

**Table 6:** KPNC counties versus KPNC for childhood and adolescent overweight and obesity in 2005 and 2010.

	Year	KPNC counties^a^	KPNC^b^
Overweight and obese	2005	36.2%	35.5%
	2010	36.5%	33.8%
Percent change^c^		+1%	−4.8%

^a^Overweight and obesity prevalence rates for KPNC counties were calculated amongst 5th, 7th, and 9th graders in 2005 and 2010 [12]. See the Methods section for the counties included.

^b^The KPNC sample consists of ages 6–19 years in 2003–2005 and 2009-2010.

^c^Percent change = percentage point difference/percent overweight and obese in 2005 or 2003–2005.
